# Secretome Analysis of the Pine Wood Nematode *Bursaphelenchus xylophilus* Reveals the Tangled Roots of Parasitism and Its Potential for Molecular Mimicry

**DOI:** 10.1371/journal.pone.0067377

**Published:** 2013-06-21

**Authors:** Ryoji Shinya, Hironobu Morisaka, Taisei Kikuchi, Yuko Takeuchi, Mitsuyoshi Ueda, Kazuyoshi Futai

**Affiliations:** 1 Graduate School of Agriculture, Kyoto University, Kyoto, Japan; 2 College of Bioscience and Biotechnology, Chubu University, Kasugai, Japan; 3 Forestry and Forest Products Research Institute, Tsukuba, Japan; Oregon State University, United States of America

## Abstract

Since it was first introduced into Asia from North America in the early 20^th^ century, the pine wood nematode *Bursaphelenchus xylophilus* has caused the devastating forest disease called pine wilt. The emerging pathogen spread to parts of Europe and has since been found as the causal agent of pine wilt disease in Portugal and Spain. In 2011, the entire genome sequence of *B. xylophilus* was determined, and it allowed us to perform a more detailed analysis of *B. xylophilus* parasitism. Here, we identified 1,515 proteins secreted by *B. xylophilus* using a highly sensitive proteomics method combined with the available genomic sequence. The catalogue of secreted proteins contained proteins involved in nutrient uptake, migration, and evasion from host defenses. A comparative functional analysis of the secretome profiles among parasitic nematodes revealed a marked expansion of secreted peptidases and peptidase inhibitors in *B. xylophilus* via gene duplication and horizontal gene transfer from fungi and bacteria. Furthermore, we showed that *B. xylophilus* secreted the potential host mimicry proteins that closely resemble the host pine’s proteins. These proteins could have been acquired by host–parasite co-evolution and might mimic the host defense systems in susceptible pine trees during infection. This study contributes to an understanding of their unique parasitism and its tangled roots, and provides new perspectives on the evolution of plant parasitism among nematodes.

## Introduction

The pine wood nematode, *Bursaphelenchus xylophilus*, is a plant parasitic nematode (PPN) and one of the most notorious forest pests in the world. The disease caused by *B. xylophilus* is called “pine wilt disease” and was first discovered in the early 20^th^ century in Japan. In 1971, *B*. *xylophilus* was shown to cause the disease [1]. Then, *B. xylophilus* spread to neighboring East Asian countries, such as China and Korea in 1982 and 1988, respectively [2]–[4], and subsequently it was found in Portugal in 1999 [5] and in Spain in 2008 [6]. It is now known that *B. xylophilus* was first described in 1934 in Louisiana, and thus originated in North America. From there it was introduced into Japan [7]. Unlike other major herbaceous PPNs, such as root knot nematodes and cyst nematodes, which infect plant roots and induce the formation of specialized feeding cells to uptake nutrients, *B. xylophilus* infects the above-ground parts of trees and quickly kills its host. Once *B. xylophilus* enters a pine tree, it migrates through the resin canals of the tree, destructively feeding on the parenchymal cells. *B. xylophilus* is also able to feed on fungal growth after the plant cells are dead.

**Figure 1:**
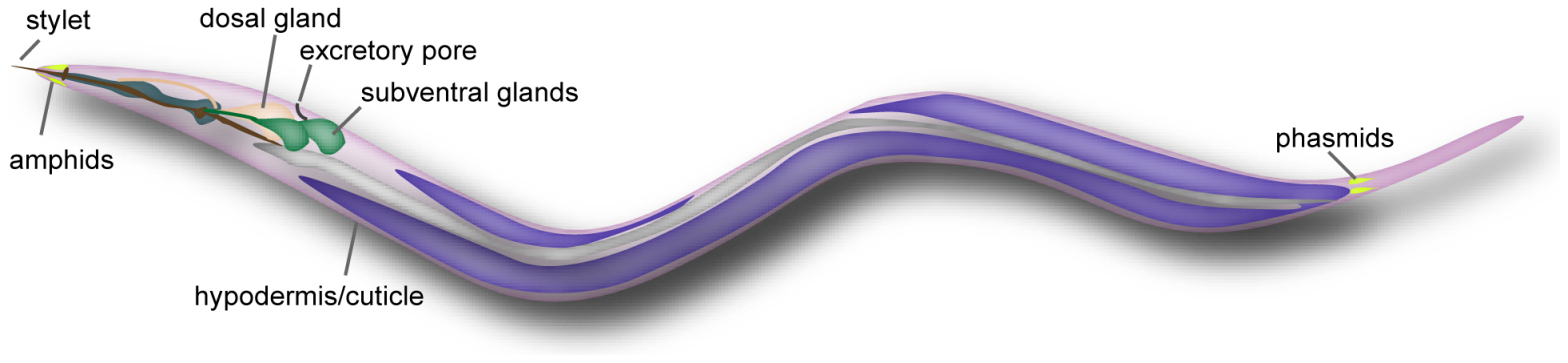
Illustration of natural openings and secretory organs of a typical plant parasitic nematode.

To obtain nutrients for development and reproduction from the cytoplasm of living cells, the fungal feeding nematodes and PPNs evolved a needle-like feeding structure, the stylet (Figure 1), as well as marked morphological and physiological modifications of the pharynx [8]. When feeding on plants and fungi, *B. xylophilus* uses the stylet to pierce the cell wall and ingest nutrients from the cytoplasm. The proteins secreted from the stylet are produced in the esophageal glands (subventral and dorsal glands) (Figure 1). Furthermore, the proteins also secreted from the hypodermis or released from natural openings of the nematode [9]. These secretions would contain cell-wall degrading enzymes and other crucial molecules for migration within plant tissues and in the interaction of the nematode with its host plant.

Owing to the importance of secreted proteins during PPN infection, these proteins have been extensively studied [10]–[13]. Early investigations of these secreted proteins were performed by biochemical approaches [14], . Around the end of 20^th^ century, rapid advances in molecular biology techniques occurred and a large number of secreted proteins were identified by expressed gene characterization [10], [16]–[18]. To date, over 100 genes encoding secreted proteins have been cloned, and the host targets and functions of several of the effector proteins have been elucidated in root knot nematodes and cyst nematodes [13]. Recently, Bellafiore *et al*. [19] performed a proteome analysis and identified 486 secreted proteins of the root knot nematode *Meloidogyne incognita*. Their study represented a significant breakthrough in the research of PPN secreted proteins, because they identified a large set of secreted proteins and because the proteins can be directly identified. There is evidence to suggest that some proteins present in secretions of nematodes may lack a classical signal peptide for secretion [20]. Therefore, proteome analysis, at present, would be the most powerful and successful approach in identifying secreted proteins.

The secreted proteins of *B. xylophilus* have also attracted the attention of many researchers as pathogenicity factor candidates in pine wilt disease [21]–[24]. However, so far only a limited number of cell-wall degrading enzymes, such as endo-β-1,4-glucanases (cellulases), β-1,3-glucanases, pectate lyase, expansin-like protein and venom allergen-like proteins [24] have been cloned and characterized. The molecular mechanisms of pathogenicity of *B. xylophilus* continue to remain controversial. This may, in part, be attributed to the limited knowledge regarding the molecules involved in the pathology as well as the lack of techniques for functional analyses. Potential pathogenic molecules from the nematodes include surface coat proteins and secretions from the stylet or other natural openings (Figure 1). So far, a profile of *B. xylophilus* surface coat proteins has been revealed by proteome analysis using the expressed sequence tag database of *B. xylophilus* [25]. Since the entire genome sequence of *B. xylophilus* was recently determined [26], it enables us to address the study of proteins on a larger scale. Thus, we conducted a large-scale identification of secreted proteins using nano-liquid chromatography coupled with tandem mass spectrometry (nanoLC-MS/MS) analysis in the present study.

The primary aim of this study was to obtain a complete picture of the proteins secreted by *B. xylophilus* using proteome analysis based on the genome sequence information [26] and to gain insight into the molecular basis of *B. xylophilus* parasitism. Furthermore, we compared the secretome profile among parasitic nematodes and reconsidered the evolution of parasitism in nematodes.

## Materials and Methods

### Nematodes

The virulent Ka4 isolate of *B. xylophilus*, which was the source for the inbred line Ka4C1 that was sequenced, was used in this study [26]. A mixed culture of the propagative forms, including 2^nd^- (J2), 3^rd^- (J3), and 4^th^-stage juveniles (J4), adults and eggs, were propagated on the fungus *Botrytis cinerea* growing on potato dextrose agar medium (Nissui-seiyaku, Tokyo, Japan) containing 100 units/ml penicillin and 100 µg/ml streptomycin at 25°C. After incubation for 10 days, the nematodes were extracted for 6 h from the culture using the Baermann funnel technique [27], and washed 10 times in sterile water containing 100 units/ml penicillin, 100 µg/ml streptomycin and 0.25 µg/ml amphotericin B.

### Preparation of the pine wood extract as a stimulant for the production of secreted proteins

The pine wood extract was prepared from nematode non-inoculated 3-year-old Japanese black pine, *Pinus thunbergii*, seedlings. The stems of the seedlings were cleaned and cut into small pieces, and 30 g of the wood pieces were soaked in 150 ml distilled water for 24 h at 4°C. Supernatant solution was then collected and passed through filter paper. To remove proteins larger than 3 kDa, which were derived from pine tissues, the extracted solution was centrifuged through an Amicon Ultra centrifugal filter (Millipore, Bedford, MA) with a 3 kDa cutoff. The solution, which passed through the membrane into the bottom of the centrifugation tube, was collected and refiltered using a 0.22-µm-pore polyvinylidene fluoride (PVDF) membrane. The solution of pine extracts thus obtained was used in the following procedure to prepare *B. xylophilus* secreted proteins for use as a stimulant.

### Preparation of secreted and whole body proteins of *B. xylophilus*

For preparation of secreted proteins, a nematode population of 1×10^7^ was soaked in 5 ml of pine wood extract on a 100 mm low attachment surface plate, EZ-BindShut dish (Iwaki, Tokyo, Japan), at 28°C for 16 h with agitation. The nematodes were then gently pelleted by centrifugation at 8 g for 5 min at 25°C. The supernatant was collected, passed through a 0.45-µm pore size hydrophilic PVDF Millex-HV syringe filter, and concentrated with Microcon YM-3 columns (Millipore) to 100 µl.

For preparation of whole *B. xylophilus* proteins, 100 µl of nematodes was solubilized in 200 µl of protein lysis buffer (9 M urea, 2% CHAPS, 20 mM Tris-HCl at pH 8.0). After one freeze (liquid nitrogen) and thaw (room temperature) cycle, the nematodes were homogenized using disposable homogenization pestle on ice. The crude lysate was then pelleted by centrifugation at 15,000 × g for 20 min at 4°C. The supernatant was collected and concentrated using the same procedure as the preparation of secreted proteins.

The protein concentrations of sample solutions were determined using a Bradford assay kit (Nacalai Tesque, Kyoto, Japan) according to the manufacturer’s protocol. A total of 45 µg of collected proteins was reduced with 10 mM Tris (2-carboxyethyl) phosphine for 60 min and alkylated with 20 mM iodoacetamide for 30 min at room temperature. After acetone precipitation, proteins were digested in 200 mM triethylammonium bicarbonate with 2.5 µg trypsin (Promega, Madison, WI) for 12 h at 37°C. Following enzymatic digestion, the peptides were applied to a proteome analysis system. Three independent biological replicates were prepared and used in the following proteome analysis.

### NanoLC-MS/MS analysis

Proteome analysis was performed by liquid chromatography (LC; Prominence nano flow system; Shimadzu, Kyoto, Japan)/mass spectrometry (MS; LTQ Velos orbitrap mass spectrometer; Thermo Fisher Scientific, Bremen, Germany). Ten microliters of proteolytic digests was injected and separated by reversed-phase chromatography using a custom-made monolithic silica capillary column, prepared from a mixture of tetramethoxysilane and methyltrimethoxysilane (300 cm long, 0.1 mm ID) as described in Motokawa *et al*. [28], at a flow rate of 500 nl/min. The gradient was provided by changing the mixing ratio of the two eluents: A, 0.1% (v/v) formic acid, and B, acetonitrile containing 0.1% (v/v) formic acid. The gradient was started with 5% B, increased to 45% B for 600 min, further increased to 95% B to wash the column, returned to the initial condition, and held for re-equilibration. The separated analytes were detected on a mass spectrometer (with full scan range 350–1500 m/z). For data-dependent acquisition, the method was set to automatically analyze the top 10 most intense ions observed in the MS scan. An ESI voltage of 2.4 kV was applied directly to the LC buffer distal to the chromatography column using a microTee (Upchurch Scientific, Oak Harbor, WA). The ion transfer tube temperature on the LTQ Velos ion trap was set to 300°C.

The mass spectrometry data were used for protein identification by Mascot software (Matrix Science, Boston, MA) with an annotated *B. xylophilus* protein database (v.1.2) obtained from GeneDB (http://www.genedb.org) [26]. MS/MS spectra were collected for secreted and whole *B. xylophilus* proteins (6,221,311 and 700,658 spectra, respectively). Since 47,835 MS/MS spectra were collected from the pine wood extract without *B. xylophilus*, these spectra were excluded and 6,173,476 MS/MS spectra were used for following *B. xylophilus* secreted protein identification analysis. The enzyme parameter was limited to full tryptic peptides with a maximum miscleavage default setting (carbamidomethylation of cysteines, +/–20 ppm for precursor ions, +/–0.6 Da for fragment ions). A decoy database was constructed to calculate the *in situ* false discovery rate (FDR) [29]. An identification filtering criteria of 1% FDR was used at the peptide level for every search. All protein matches were required to be detected in at least two of three biological replicates. Proteins with single peptide hits were limited to unique peptide sequences not matched in any higher ranked proteins.

To compare the relative abundance of proteins between secretome and whole *B. xylophilus* proteins, we used the spectral counting method described in Bellafiore *et al*. [19]. Total spectral counts were used for normalization of the spectral counts from each protein. The normalized spectral count ratio of each protein (secretome/whole *B. xylophilus* proteins) was used as the index of protein abundance in secretome and whole *B. xylophilus* proteins.

### Comparison of secretome profiles among parasitic nematodes based on OrthoMCL cluster analysis and Gene Ontology (GO) analysis

To detect the putative orthologs in the secretomes of other parasitic nematodes, we performed an OrthoMCL cluster analysis, using the default settings (E-value cutoff 1e-5 and percent identity 50%), on the 1,515 secreted proteins of *B. xylophilus* against the secreted proteins of two other parasitic nematodes, *M. incognita* and *Brugia malayi* [30]. The secretome sequence lists of *M. incognita* and *B. malayi* were obtained from Bellafiore *et al*. [19] and Bennuru *et al*. [20], respectively. In *B. xylophilus*, GO annotation made by Kikuchi *et al*. [26] was used. GO annotations of the secretomes of *M. incognita* and *B. malayi* were performed in the same manner as the annotation for *B. xylophilus*. In brief, GO annotations were initially derived using Blast2GO software [31] based on the BLAST match against NCBI non-redundant proteins with an E-value cutoff of 1e-10. The GO annotations were also performed with InterProScan [32], and then these annotation data were merged together within the Blast2GO.

### Detection of peptidases and peptidase inhibitors

To detect putative peptidases (also termed proteases or proteinases) and peptidase inhibitors, a MEROPS batch BLAST search was performed [33]. A total of 1,515 secreted protein sequences were subjected to the MEROPS BLAST search and classified into detailed MEROPS peptidase or peptidase inhibitor families (E-value cutoff of 1e-5) [33]. To compare the peptidases and peptidase inhibitors in the secretome of *M. incognita* and *B. malayi*, the same procedures were performed against each secretome profile.

### Detection of plant/fungal cell-wall degrading enzymes

The CAZymes Analysis Toolkit [34] was used to detect *B. xylophilus* carbohydrate active enzymes (CAZymes) based on the CAZy database. An annotation method “based on association rules between CAZy families and Pfam domains” was used with an E-value threshold of 0.01, a bitscore threshold of 55 and rule support level 40. The putative plant/fungal cell-wall degrading enzymes were manually selected from the CAZymes according to Kikuchi *et al*. [26]. In addition, only for expansin, which would be involved in cell-wall modification, the candidate proteins were manually identified based on the InterProScan based annotation data.

### Detection of proteins acquired by horizontal gene transfer

The potential horizontal gene transfers (HGT) specifically into *B. xylophilus* were manually identified based on the data in Kikuchi *et al*. [26]. Kikuchi *et al*. [26] performed BLASTP analysis to compare predicted *B. xylophilus* protein sequences against the NCBI nr database, producing a candidate set of horizontally transferred genes that had significant BLAST hits (E-value <1e-10) to organisms other than nematodes and no significant BLAST hits (E-value ≤1e-5) to any nematode sequence except for genes from Aphelenchoidea. For each of HGT candidates, amino acid sequence data was extracted from the NCBI database, aligned using Muscle and generated Maximum Likelihood phylogenetic trees. The candidates supported by phylogenetic evidence were identified as HGT genes [26].

### Detection of potential *B. xylophilus* proteins that mimic the host plant defense system

To find proteins in the *B. xylophilus* secretome that mimic those of the host plant, we performed BLASTP analysis to compare *B. xylophilus* protein sequences against the NCBI nr database. The proteins that had a significant top BLAST hit to plant proteins (E-value <1e-4) and no significant BLAST hits (E-value <0.1) to any nematode sequence were identified as potential proteins that mimic the host plant defense system.

## Results and Discussion

### Extraction and stimulation of the secreted proteins of *B. xylophilus*

In the preliminary test, the efficiency of three potential stimulants, 5-methoxy-N, N-dimethyl tryptamine oxalate (DMT), resorcinol, and pine wood extract, were examined for efficacy to induce the secretion of proteins of *B. xylophilus*. DMT and resorcinol had been reported as stimulants of protein secretion in other PPNs [35], [36]. The pine wood extract showed the highest efficacy in induction of secreted proteins of the four test solutions (0.4% resorcinol, 400 µg/ml DMT, pine wood extract and phosphate buffered saline), and the efficacy was not lost even when high molecular weight (> 3,000 Da) molecules were removed from the extract (data not shown). This result suggested that the water soluble low molecular weight compounds in the pine extract could induce the secretion of proteins. Furthermore, three classes of pine seedlings, those that had been inoculated with nematodes three days prior (early stage of infection), those that had been inoculated with nematodes 2 weeks prior (late stage of infection), and nematode free (non-infested), were prepared and treated with *B. xylophilus* to determine the optimal conditions of pine seedlings as a stimulant. As a result, a significant decrease in the efficacy of secretion induction was observed only in the pine seedlings in the late stage of infection (data not shown). Since the mixed stages of nematodes in the propagative stages, which are the stages for propagation in the pine tree and the cause of the pine wilt, were used in this study, the secreted *B. xylophilus* proteins used for this proteome analysis should include those in the early stage of infection. In order to exclude the possibility of potential contamination of the pine-derived proteins in the *B. xylophilus* secretome, we employed the pine wood extract not infected with nematodes as a control. No proteins were detected in gels by silver staining, but using mass spectrometry, 47,835 MS/MS spectra and 15 peptides were found and overlapped with the *B. xylophilus* secretome samples. These proteins were derived from the pine extract and were excluded in the protein identification analysis.

### Identification of the *B. xylophilus* secretome

In this secretome analysis, 1,515 distinct secreted proteins of *B. xylophilus* were identified. Kikuchi *et al*. [26] predicted that the *B. xylophilus* genome includes 18,074 protein-coding genes, meaning that at least 8.4% of the total *B. xylophilus* proteins are secreted. The complete list of identified secreted proteins is given in a concise form as supplemental information (Table S1, Dataset S1). These include all secreted proteins previously reported by Kikuchi *et al*. [22], [23], [37], [38] and Lin *et al*. [24] indicating that the results obtained here both confirm and extend previous studies. The majority of secreted proteins of *B. xylophilus* (72.0%) were identified by detecting two or more peptides. Among 1,515 proteins identified in this study, 510 proteins were not able to be annotated. The draft genome data indicated that *B. xylophilus* produces 3,365 predicted secreted proteins in total. In this study, 625 of a total of 1,515 secreted proteins had putative secretion signal sequence(s), and therefore, the remaining 58.7% may have unknown secretory signals or may be secreted through non-classical secretory pathways. The proportion of the identified proteins bearing a secretion signal in this study is similar to a previous secretome study on the filarial nematode *B. malayi* [20] and is higher than that of *M. incognita* [19], suggesting that the secreted proteins lacking known secretion motifs are common to the nematodes.

**Figure 2:**
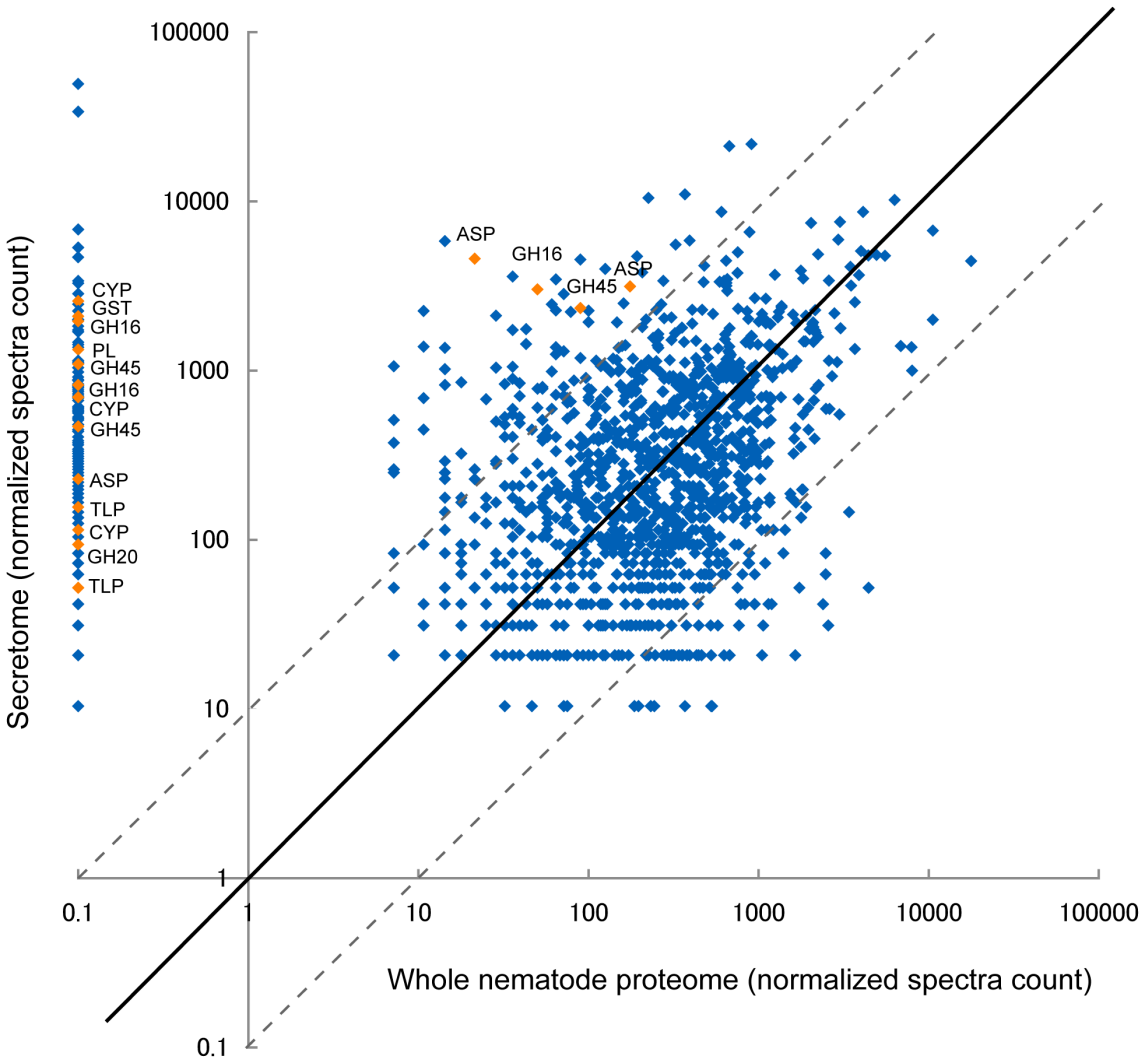
Relative abundance of secreted proteins compared to proteins from the whole ***Bursaphelenchus xylophilus*** **lysate.**

The whole *B. xylophilus* proteins were also identified to serve as a control for potential contamination of the secretome by somatic proteins (Table S2). The relative abundance of proteins between secreted proteins and whole *B. xylophilus* proteins were shown in Table S1 and Figure 2. Approximately 27% (407) of the secreted proteins were at least 10-fold more abundant than the whole *B. xylophilus* proteins. On the other hand, only 4% (66) of the secreted proteins were 10-fold less abundant than the whole *B. xylophilus* proteins. Most of proteins discussed in detail (cell-wall degrading enzyme, HGT, host mimicry etc.) were significantly less abundant or absent in the whole *B. xylophilus* proteome (Figure 2). These results provide further evidence that they are indeed secreted. The abundant proteins in the whole *B. xylophilus* proteins may be contaminations, but there is a possibility that these proteins were truly secreted.

### Comparing the secretome profiles of parasitic nematodes and the expansion of peptidases in the *B. xylophilus* secretome

**Figure 3:**
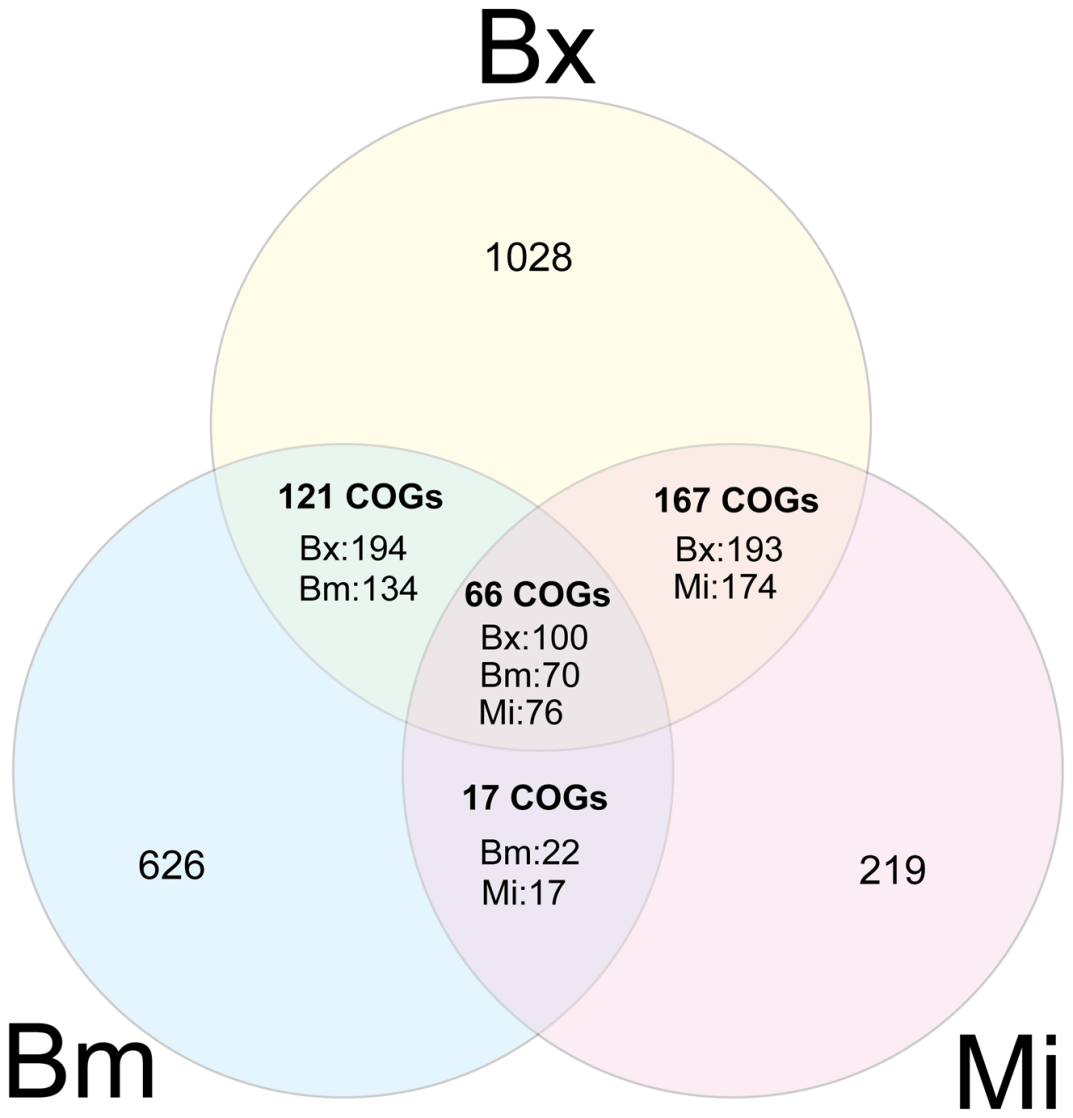
Venn diagram showing the distribution of shared gene families among the nematode secretomes. Bold numbers indicate the clusters of orthologous groups (COGs). Non-bold numbers indicate the genes in each cluster. Bx: *Bursaphelenchus xylophilus*; Mi: *Meloidogyne incognita*; Bm: *Brugia malayi*.

**Figure 4:**
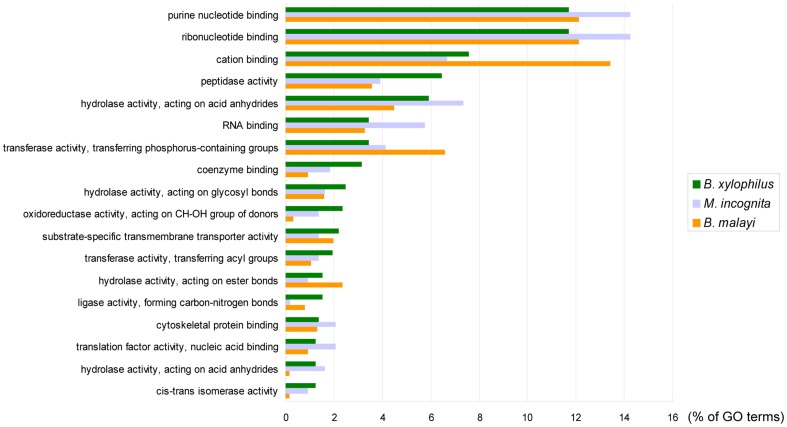
Distribution of molecular functions in Gene Ontology terms (Level 4) in the nematode secretomes.

A comparison of secretome profiles among three different parasitic nematodes, *B. xylophilus*, *M. incognita* and *B. malayi*, revealed the difference of secretomes with each other. The results of an OrthoMCL cluster analysis showed that 1,028 proteins were specific to *B. xylophilus*. The three nematodes shared 66 clusters of orthologous groups (COGs), including 100 *B. xylophilus* proteins (Figure 3, Table S3). *B. xylophilus* and *M. incognita* shared 233 COGs (293 proteins of *B. xylophilus* have hits to 250 proteins of *M. incognita*), and this was equivalent to 51.4% of the secreted proteins of *M. incognita* (Figure 3, Table S4). *B. xylophilus* and *B. malayi* shared only 23.9% of the secreted proteins of *B. malayi* (294 proteins of *B. xylophilus* have hits to 204 proteins of *B. malayi*) (Figure 3, Table S5). Furthermore, a GO analysis among nematodes clearly showed the expansion of peptidases in the secretome of *B. xylophilus* (Figure 4). In particular, a large number of cysteine and aspartic peptidases were detected in *B. xylophilus* (Table 1). The genome sequence also revealed that *B. xylophilus* has a large number of predicted peptidase genes [26]. The expansion of peptidases was also reflected in the secretome of *B. xylophilus*. As a result of a MEROPS BLAST analysis, a total of 161 secreted peptidases were identified in the *B. xylophilus* secretome (Table 1). The percentage of peptidases in the *B. xylophilus* secretome was 10.6%, whereas in *M. incognita* and *B. malayi* there were 6.4% and 5.2%, respectively. As a result of peptidase classification, C1A (papain) family cysteine peptidases and A1A (pepsin) family aspartic peptidases were both identified as major secreted peptidase groups by MEROPS BLAST. In addition, a GO analysis among nematodes also indicated the expansion of proteins with nucleotide binding functions in the *M. incognita* secretome and the expansion of proteins with cation (metal) binding functions in the *B. malayi* secretome. *M. incognita* induces the differentiation of root cells into specialized feeding cells called ‘giant cells’. It has been suggested that the secreted proteins that target host nuclei manipulate nuclear functions of the cells and induce giant cells [19], [39]. Therefore, the expansion of proteins with nucleotide binding functions would be a key event toward the establishment of a unique feeding strategy and highly specialized plant parasitism. In the secretome analysis of *M. incognita*, only the J2 stage was used [19], although the mixed stages of the propagative forms were used in *B. xylophilus*. It is possible that the differences in secretome profiles and the number of secreted proteins identified are due to the different life stages used in the secretome analysis. However, the J2 stage in *M. incognita* is an infective stage to host plants. Similarly, the propagative forms of *B. xylophilus* are stages that directly contact the host tree and cause disease (Figure S1). Therefore, it is appropriate to compare the J2 stage of *M. incognita* and the mixed stages of the propagative forms of *B. xylophilus* to compare their plant parasitisms, because there is no evidence that only a specific stage in the propagative forms of *B. xylophilus* is responsible for the disease.

**Table 1:** Summary of secreted peptidases in *Bursaphelenchus xylophilus* and other nematodes.

	*B. xylophilus*	*M. incognita^a^*	*B. malayi^b^*
Asp	31 (2.0)	0 (0)	1 (0.1)
Cys	53 (3.5)	7 (1.4)	11 (1.3)
Metal	34 (2.2)	11 (2.3)	24 (2.8)
Ser	30 (2.0)	4 (0.8)	5 (0.6)
Thr	13 (0.9)	9 (1.9)	3 (0.4)
Total	161 (10.6)	31 (6.4)	44 (5.2)

According to Merops, the proteins were classified by catalytic types: aspartic (Asp), cysteine (Cys), metallo (Metal), serine (Ser) and threonin (Thr).

The percentages of the proteins out of the total secreted proteins identified by proteome analysis are shown in parentheses.

a, b These values are calculated from the secretome data of Bellafiore *et al*. [19] and Bennuru *et al*. [20], respectively.

A phylogenetic tree of the C1A (papain) family cysteine peptidases showed that the C1A family cysteine peptidases have undergone the greatest expansion via gene duplication without HGT from ancestral cysteine peptidase genes (Figure S2). However, a phylogenetic tree of the A1A (pepsin) family aspartic peptidases indicated that HGT is a primary driving force in the expansion of A1A family aspartic peptidases (Figure S3). Why does *B. xylophilus* increase the number of peptidases by gene duplication and HGT? Peptidases could act in at least two ways to assist the survival of *B. xylophilus*: they may be required for nutritional purposes or utilized to degrade proteins in the plant cell wall, allowing for the spread of nematodes or the defeat of host defenses. One explanation for nutritional purposes is that *B. xylophilus* can uptake nutrients from a variety of food sources. *B. xylophilus* can feed both on fungi and living plant cells. More correctly, *Bursaphelenchus* spp. were originally a mycophagous group [40] that has adapted to feeding on living plant tissues. In nature, when living tree cells are no longer available after tree death, *B. xylophilus* feeds and reproduces on the fungal hyphae growing along the resin canals. On the other hand, *Meloidogyne* spp. are obligate plant parasites, and they can uptake nutrients only from host plant cells. Kikuchi *et al.* [26] showed that both *M. incognita* and *Meloidogyne hapla* have fewer putative peptidase genes in their genome than other nematodes. Furthermore, very few peptidases were detected in the secretome of *M. incognita* (Table 1). Taking into account the evolutionary development of nematodes (Figure S4) [41], [42], *B. xylophilus* would gain peptidase genes by gene duplication and HGT, whereas the obligate plant parasitic groups, such as *M. incognita*, would lose many of the peptidase genes as they adapted to each food source and/or life cycle. In the PPNs, very few reports regarding secreted peptidases have been presented so far [19], [43]. In addition, there are no reports of a functional analysis of the secreted peptidases in plant–nematode interactions. However, in the animal parasitic and entomopathogenic nematodes, there are many examples of secreted peptidases that are involved in tissue invasion/migration [44]–[50]. It has been indicated that these peptidases contribute to host specificity, host range and virulence [48], [51]. Therefore, it is quite likely that the secreted peptidases of *B. xylophilus* have a key role in its successful parasitism of pine trees. However, further functional analyses are needed to determine an exact role of the peptidases in host–parasite interactions.

### Cell-wall degrading enzyme

Enzymes involved in the carbohydrate metabolic process, including glycoside hydrolases (GH), carbohydrate esterases (CE), glycosyl transferases (GT), carbohydrate-binding modules (CBM) and polysaccharide lyases (PL), are referred to as carbohydrate active enzymes. Some of these are also known as cell-wall degrading enzymes. In total, 65 putative carbohydrate active enzymes were identified in the secretome of *B. xylophilus* in this study (Table S6). Furthermore, we manually detected four expansin proteins based on the InterProScan based annotation data. Among them, we detected 28 putative plant/fungal cell-wall degrading enzymes based on Kikuchi *et al*. [26] (Table 2). It has been suggested that GH45 cellulase, pectate lyase and expansin proteins are the major plant cell-wall degrading enzymes of *B. xylophilus*, because the principal carbohydrates of the primary cell wall of plant are cellulose, hemicellulose and pectin [52]. GH16 β-1,3-glucanase and chitinases (GH18 and GH20) have also been suggested to play important roles in fungal cell-wall degradation by the basically mycophagous *B. xylophilus* because β-1,3-glucan and chitin are main components of the fungal cell wall [53]. The results of this study are the first evidence of the secretion of these enzymes by *B. xylophilus*. The profiles of the cell-wall degrading enzymes of *B. xylophilus* also suggested that *B. xylophilus* has quite different mechanisms of cell-wall degradation from *M. incognita*, because no putative fungal cell-wall degrading enzymes were identified in the secretome of *M. incognita* [19]. Such a difference would reflect the difference in their food sources. *B. xylophilus* can use both plant cells and fungal ones as food sources, while most other obligate PPNs feed on only plants. Therefore, the differences in cell-wall degrading enzymes would allow the unique life style of the facultative PPN *B. xylophilus*.

**Table 2:** Potential plant/fungal cell-wall degrading enzymes in the *Bursaphelenchus xylophilus* secretome.

Family	Substrate	Total No.	GeneDB protein ID	Top BLAST hit
GH16	1,3-glucan	5	BUX.s00705.10	beta-1,3-endoglucanase
			BUX.s01066.142	beta-1,3-endoglucanase
			BUX.s01066.143	beta-1,3-endoglucanase
			BUX.s01066.145	beta-1,3-endoglucanase
			BUX.s01066.63	beta-1,3-endoglucanase
GH18	chitin	5	BUX.s00422.469	chitinase family member (cht-1)
			BUX.s01038.115	endochitinase
			BUX.s01038.116	chitinase I
			BUX.s01092.2	chitinase
			BUX.s01661.27	chitinase I
GH20	chitin	4	BUX.s00252.68	beta-n-acetylhexosaminidase
			BUX.s00252.69	beta-n-acetylhexosaminidase
			BUX.s00336.26	beta-n-acetylhexosaminidase
			BUX.s00422.475	CBR-HEX-3 protein
GH45	cellulose	7	BUX.s00036.112	beta-1,4-endoglucanase
			BUX.s00036.113	beta-1,4-endoglucanase
			BUX.s00119.43	beta-1,4-endoglucanase
			BUX.s00119.44	beta-1,4-endoglucanase
			BUX.s00397.15	beta-1,4-endoglucanase
			BUX.s00397.16	beta-1,4-endoglucanase
			BUX.s01038.221	beta-1,4-endoglucanase
PL	pectin	3	BUX.s00460.341	pectate lyase
			BUX.S01259.21	pectate lyase
			BUX.S01661.75	pectate lyase
EXPN	-	4	BUX.s01281.215	expansin-like protein
			BUX.s01281.223	expansin-like protein
			BUX.s01281.227	expansin-like protein
			BUX.s01281.230	expansin-like protein

Each candidate was classified by glycoside hydrolase families based on the carbohydrate-active enzymes (CAZy) database.

PL: Pectate lyase; EXPN: Expansin.

### The secreted proteins of *B. xylophilus* acquired by horizontal gene transfer

The *B. xylophilus* genome includes some genes acquired from other organisms by HGT [25]. Kikuchi *et al*. [26], supported by phylogenetic evidence, indicated that a total of 24 genes were putatively horizontally transferred genes. In this study, we identified 16 of the 24 (66.7%) putatively horizontally transferred proteins in the secretome of *B. xylophilus*. There were three aspartic endopeptidase, one cysteine peptidase inhibitor, seven GH45 family members, and five GH16 β-1,3-glucanases (Table 3). They were acquired from ascomycete fungi and bacteria. Since the percentage of secreted proteins in the total predicted proteins was 8.6%, the ratio (66.7%) of secreted proteins to whole proteins acquired by HGT was significantly higher. Since these proteins acquired by HGT are essential for survival and give fitness advantages to *B. xylophilus*, they would be positively selected for over evolutionary time.

**Table 3:** Putatively horizontally transferred proteins, supported by phylogenetic evidence, secreted from *Bursaphelenchus xylophilus*.

GeneDB protein ID	Annotation	Likely source
BUX.s00460.56	aspartic peptidase	ascomycete fungi
BUX.s01281.82		
BUX.s00110.147		
BUX.s00351.346	cystein peptidase inhibitor	gamma-proteobacteria
BUX.s00119.43	GH45 hydrolase	ascomycete fungi
BUX.s00397.15		
BUX.s00119.44		
BUX.s00397.16		
BUX.s01038.221		
BUX.s00036.112		
BUX.s00036.113		
BUX.s01066.145	GH16 β-1,3-glucanase	gamma-proteobacteria
BUX.s01066.63		
BUX.s01066.142		
BUX.s00705.10		
BUX.s01066.143		

Likely source indicates sister-taxon or sister-taxa of the gene copy in the phylogeny.

### Evasion from the host defense response

It is worth highlighting proteins related to evasion from the host defense response, because continuous excess ROS generation is a distinctive characteristic of pine wilt disease and is suggested to be an important factor in the development of disease symptoms [54], [55]. Thus, the effective anti-oxidant ability is of critical importance in establishing the infection. In this study, only the proteins that showed anti-oxidant activity, catalase activity or superoxide dismutase activity in the molecular functions of the GO analysis were regarded as anti-oxidant proteins. As a result, a total of 12 anti-oxidant proteins were identified in the secretome of *B. xylophilus* (Table 4), including peroxiredoxin, catalase, glutathione peroxidase, nucleoredoxin-like protein, superoxide dismutase, and thioredoxin. In addition, four homologs of glutathione-s-transferases, well known as a detoxifying enzyme, were identified. *B. xylophilus* also accumulates anti-oxidant and detoxifying enzymes on the body surface [25]. *B. xylophilus* mainly inhabits the resin canals and rays of the host pine tree. The resin canals are filled with several terpenes including thick and sticky ones. Furthermore, in the early stage of a *B. xylophilus* infection, abundant ROS is produced in the pine tree [56]. Therefore, the resin canal would be an extremely severe environment for *B. xylophilus* [57]. The secreted anti-oxidant and detoxifying enzymes would play a pivotal role in protecting *B. xylophilus* itself from oxygen free radicals and toxic compounds in the pine tree.

**Table 4:** Anti-oxidant and detoxifying enzyme proteins that are secreted from *Bursaphelenchus xylophilus*.

GeneDB protein ID	Annotation	Representative target ROS
BUX.s01109.377	catalase	H_2_O_2_
BUX.s00139.134	glutathione peroxidase	H_2_O_2_, LOOH
BUX.s00422.418		
BUX.s01109.624	superoxide dismutase (Cu–Zn)	O_2_^−^
BUX.s01438.70	superoxide dismutase (Mn)	O_2_^−^
BUX.s00961.40	glutathione s-transferase 1	LOOH
BUX.s00961.42		
BUX.s00647.119	glutathione s-transferase 3	LOOH
BUX.s00647.122		
BUX.s00351.179	peroxiredoxin	H_2_O_2_
BUX.s01109.415		
BUX.s00116.938	thioredoxin	Other
BUX.s01653.267		
BUX.s01092.9	nucleoredoxin-like protein 2	Other
BUX.s01102.40		
BUX.s01513.347		

In addition to ROS generation in the plant defense system, plant peptidases also play key roles against pathogens and pests. The MEROPS BLAST analysis detected 47 putative peptidase inhibitors in the *B. xylophilus* secretome (Table 5). The number of secreted peptidase inhibitors of *B. xylophilus* was significantly greater than other parasitic nematodes. In particular, the inhibitors in subfamily I25B and I29 were highly expanded in *B. xylophilus* secretome (Table 5). Inhibitor family I25B and I29 primarily contain inhibitors of cysteine peptidases (C1A). Some I25B family proteins also inhibit legumain (C13). It is known that the families of cysteine peptidases C1A and C13 play key roles in various physiological phenomena in plants [58]–[60], and they are involved in the regulation of the plant defense system [61]. A role in defense against pathogens by executing programmed cell death using the caspase activity observed for these cysteine peptidases has been proposed [62]. Therefore, the main role of the expanded I25B and I29 peptidase inhibitors in the *B. xylophilus* secretome could be to battle against host plant cysteine peptidases. Recently, Hirao *et al*. [63] revealed that the expression of peptidase genes in a susceptible family of Japanese black pines was induced more quickly and more significantly during *B. xylophilus* infection than in a resistant family. Such an overexpression of peptidase genes was one of the most intense reactions in the *B. xylophilus* infection at the gene expression level. Therefore, the interactions of peptidases with their substrates and inhibitors must be one of the most important molecular battle-fields at the *B. xylophilus*-pine interface. Since the number of secreted peptidase inhibitors in *B. xylophilus* was significantly greater than in other parasitic nematodes, the expansion of peptidase inhibitors could result from the competitive co-evolution of *B. xylophilus* and pines or between a close ancestor of *B. xylophilus* and its host.

**Table 5:** Summary of secreted peptidase inhibitors in *Bursaphelenchus xylophilus* and other nematodes.

Family (Type of inhibitor)	*B. xylophilus*	*M. incognita^a^*	*B. malayi^b^*
I02 (aprotinin)	5	0	3
I04 (alpha-1-peptidase inhibitor)	0	1	4
I08 (chymotrypsin/elastase inhibitor)	2	0	2
I25B (ovocystatin)	10	1	1
I29 (cytotoxic T-lymphocyte antigen-2 alpha)	23	0	0
I31 (equistatin inhibitor unit 1)	1	0	0
I32 (survivin)	0	0	1
I33 (aspin)	1	0	0
I39 (alpha-2-macroglobulin)	1	0	2
I51 (serine carboxypeptidase Y inhibitor)	2	1	2
I63 (pro-eosinophil major basic protein)	2	0	0
Total	47	3	15

The inhibitor types were classified according to Merops.

a, b These values are calculated from the secretome data of Bellafiore *et al*. [19] and Bennuru *et al*. [20], respectively.

### Potential *B. xylophilus* proteins that mimic host plant defense systems

In the *B. xylophilus* secretome, we identified three secreted proteins that have high sequence similarity to plant proteins, while no similar proteins were detected in other nematode species (E-value <0.1). These included two putative thaumatin-like proteins and a cystatin-like peptidase inhibitor (Table 6). In plants, thaumatin-like proteins are known as pathogenesis-related protein 5 (PR-5), and they have an antifungal activity that acts by permeabilizing fungal membranes [64]. Furthermore, they also appear to function by binding and hydrolyzing β-1,3-glucans [65], or inhibiting fungal xylanases [66]. Moreover, it was reported that thaumatin in plants has a possible role in activating other plant defense pathways, including phenylpropanoid and phytoalexin production [67]. So far, thaumatin-like proteins have been discovered in a wide range of organisms, including plants, nematodes [68], insects [69], and fungi [70], [71]. However, the two thaumatin-like proteins in the secretome of *B. xylophilus* were more like those of plants than those of other nematodes. Surprisingly, the thaumatin-like proteins of *B. xylophilus* showed the highest level of similarity to the proteins of pine tree (the genus *Pinus*).

**Table 6:** Potential molecular mimicry proteins against host plants in *Bursaphelenchus xylophilus*.

GeneDB protein ID	Annotation	Top Blast hit plant	e-value	Top Blast hit nematode	e-value
BUX.s00036.92	thaumatin-like protein	*Pinus teada*	4.28E-13	protein THN-1 [*Caenorhabditis elegans*]	0.83
BUX.s00036.89		*Pinus monticola*	8.55E-05	protein THN-3 [*Caenorhabditis elegans*]	> 10
BUX.s00351.347	cysteine proteinase inhibitor	*Medicago truncatula*	1.00E-05	ani s 4 allergen [*Anisakis simplex*]	0.35

As mentioned above, cystatin mainly inhibits peptidases belonging to the C1 (papain family) and C13 (legumain family) peptidase families. Multiple roles have been attributed to cystatins in plants, including the control of endogenous cysteine peptidases, seed development, and programmed cell death [72]–[74]. Furthermore, plant cystatins also play a significant role in plant defenses against pathogenic microbes, herbivorous arthropods, and PPNs [73], [75], [76]. The cystatin-like peptidase inhibitor in the secretome of *B. xylophilus* was most similar to a cystatin-like peptidase inhibitor of the herbaceous plant *Medicago truncatula*. Molecular mimicry is one of the well-known strategies for parasite host defense system evasion and host manipulation. One well-known example of molecular mimicry is the secretion of proteins by animal parasitic nematodes [77]. The helminth parasite *B. malayi* secretes homologs of the human cytokine macrophage migration inhibitory factor (MIF) [78]. *B. malayi*-encoded MIFs have the same effect on human monocytes as the mammalian MIF. Molecular mimicry in PPNs has also been reported. Examples of these molecules include the CLAVATA3/ESR-like (CLE) peptides [79]. The nematode CLEs may play roles through molecular mimicry in the regulation of certain root meristematic cells that are essential for the establishment of feeding sites in the host, a critical step for nematode parasitism of plants [79]–[81]. In addition, Bellafiore *et al*. [19] identified several secreted proteins of the root knot nematode *M. incognita* that are homologous to plant proteins by proteome analysis, although the function of these proteins still remains unclear. In *B. xylophilus*, there has been no report of molecular mimicry until this finding. Recently, Hirao *et al*. [63] revealed that the expression of antimicrobial peptides and putative pathogenesis-related genes (*e.g.*, PR-1b, 2, 3, 4, 5, and 6) was much higher in susceptible trees than in resistant trees, irrespective of the time after nematode infection. In particular, the genes PR-5 (thaumatin) and PR-6 (peptidase) were significantly overexpressed in susceptible trees during *B. xylophilus* infections. That is, the molecular mimicry candidates in *B. xylophilus* were expressed concurrently with the most noteworthy proteins in susceptible pine trees. This correspondence is an intriguing and compelling finding. Although it is difficult to determine the evolutionary origin of the potential mimicry proteins of *B. xylophilus*, these molecular mimicry proteins could have been acquired by host–parasite co-evolution. Only about 100 years have passed since the first report of pine wilt disease. From this viewpoint, co-evolution could have occurred through an interaction with native pine species in North America. The native pine trees should have evolved to overcome these molecules from the parasites during host–parasite co-evolution. In contrast, non-native pine species to North America would not have evolved with respect to resistance against these mimicry molecules of newly emerged parasites and would be highly susceptible to their damage. Therefore, the abnormal responses in susceptible pine trees (mostly non-native pine species) might be induced by mimicry molecules secreted by *B. xylophilus*.

## Concluding Remarks

This study provides a comprehensive profile of the secretome of *B. xylophilus*, comprising a large array of proteins, many of which have been shown to be important in nutrition, invasion/migration in host trees, modulating the host immune evasion, and potentially promoting their survival. In addition, many “hypothetical proteins” were detected by LC-MS/MS-based secretome analysis. In this secretome analysis the application of high-throughput proteomic techniques allowed the identification of low abundance proteins that might not be detected using gel electrophoresis-based methods. Typical SignalP analyses would not account for 58.7% of the secreted proteins identified here, and this suggests that new methods for these predictions need to be properly tailored to (parasitic) nematodes. Furthermore, the most important finding in this study is that *B. xylophilus* secretes a large number and various types of peptidases and peptidase inhibitors. The theories of gene duplication suggest that ancestral genes possessed multiple functions prior to duplication that were then preserved and refined in the duplicated genes by subsequent positive selection to evolve paralogs with distinct functions [82], [83]. Therefore, it is quite likely that these secreted peptidases and peptidase inhibitors, exceptionally expanded, are essential molecules for parasitism by *B. xylophilus*. As for the cysteine peptidase inhibitors, we revealed not only their expansion but also the molecular mimicry potential to host pines. Taking into consideration that *B. xylophilus* is an exotic pathogen in Asia and Europe, these potential host mimicry proteins that co-evolved in relation to the native pine species of North America might be crucial molecules in the pathogenicity of *B. xylophilus* against the non-native susceptible pine trees.

The comparative functional analysis of the *B. xylophilus* secretome and the *M. incognita* secretome showed the unique expansion of peptidases and nucleotide binding proteins in *B. xylophilus* and *M. incognita*, respectively, although 51.4% of the secreted proteins of *M. incognita* were shared with those of *B. xylophilus*. These unique expanded molecules seem critical to each unique parasitism. In the study of pine wilt disease, researchers have sought molecules similar to or homologous with effector molecules of obligate PPNs for a long time. However, such an approach would not be effective for identifying crucial molecules in, and for understanding the parasitism of *B. xylophilus* because the strategy and the evolutionary processes of plant parasitism would be significantly different. The secretome information of *B. xylophilus* will aid us in addressing the issue of parasitism by *B. xylophilus* separately from those of the obligate PPNs. Future research that focuses on these unique secreted proteins of *B. xylophilus* will be useful in revealing mechanisms of pine wilt disease and in developing novel control strategies for pine wilt disease.

## Supporting Information

Figure S1 **Life cycle of** ***Bursaphelenchus xylophilus*** **.** The black arrows show *B. xylophilus* development cycle. The solid black arrows and dashed black arrows show the propagative cycle in pine trees (propagative forms) and that for transmission to new host trees by beetle vectors (dispersal forms), respectively. After invading healthy trees the forth-stage dispersal juvenile (DIV) of *B. xylophilus* molts to become the adult, and the propagative forms of *B. xylophilus* feed on the parenchymal cells in the resin canals. *B. xylophilus* is also able to feed on fungal growth after the plant cells are dead.
(TIF)

Figure S2 **Phylogenetic relationships of the C1A (papain) family of cysteine peptidase secreted from** ***Bursaphelenchus xylophilus*** **.** A multiple alignment of 205 aa was analyzed by Muscle and the phylogenetic tree was built using the maximum likelihood method in MEGA5 based on the JTT model with 1,000 bootstrap replicates. Bootstrap values more than 50% were shown in the tree. Four proteins (BUX.s00713.538, BUX.s0813.53, BUX.s0983.4, and BUX.s01063.86) with short lengths or with long branches in the preliminary tree were removed from the analysis. The scale bar indicates number of amino acid changes per site.
(TIF)

Figure S3 **Phylogenetic relationships of the A1A (pepsin) family of aspartic peptidase secreted from** ***Bursaphelenchus xylophilus*** **.** A multiple alignment of 338 aa was analyzed by Muscle and the phylogenetic tree was built using the maximum likelihood method in MEGA5 based on the JTT model with 1,000 bootstrap replicates. Bootstrap values more than 50% were shown in the tree. Five proteins (BUX.c03104.1, BUX.s01038.154, BUX.s01038.155, BUX.s00460.238, and BUX.s01150.38) with short lengths or with long branches in the preliminary tree were removed from the analysis. The scale bar indicates number of amino acid changes per site. *HGT indicates that the protein was acquired from other organism via horizontal gene transfer. These were selected based on the data of Kikuchi *et al*. [26].
(TIF)

Figure S4 **Schematic representation of the evolution of plant parasitism and the phylogenetic relationships of nematodes.** The figure is adapted from Blaxter *et al*. [41] (major clades) and van Megen *et al*. [42] (minor clades).
(TIF)

Table S1 **Proteins identified in the secretome of** ***Bursaphelenchus xylophilus*** **.**
(XLS)

Table S2 **Proteins identified in the whole body lysate of** ***Bursaphelenchus xylophilus.***
(XLS)

Table S3 **The list of clusters of orthologous groups and genes shared among the secretomes of** ***Bursaphelenchus xylophilus*** **(Bx),** ***Meloidogyne incognita*** **(Mi) and** ***Brugia malayi*** **(Bm).**
(XLS)

Table S4 **The list of clusters of orthologous groups and genes shared between the secretome of** ***Bursaphelenchus xylophilus*** **(Bx) and** ***Meloidogyne incognita*** **(Mi).**
(XLS)

Table S5 **The list of clusters of orthologous groups and genes shared between the secretome of** ***Bursaphelenchus xylophilus*** **(Bx) and** ***Brugia malayi*** **(Bm).**
(XLS)

Table S6 **The list of carbohydrate active enzymes in the secretome of** ***Bursaphelenchus xylophilus*** **.**
(XLS)

Dataset S1 **Protein datasets used for secretome identification.**
(TXT)
