# Supplementary figures and images for: Secretome Analysis of the Pine Wood Nematode Bursaphelenchus xylophilus Reveals the Tangled Roots of Parasitism and Its Potential for Molecular Mimicry
Source: PLoS One. 2013 Jun 21;8(6):e67377. doi: 10.1371/journal.pone.0067377 (PMC3689755; doi:10.1371/journal.pone.0067377)

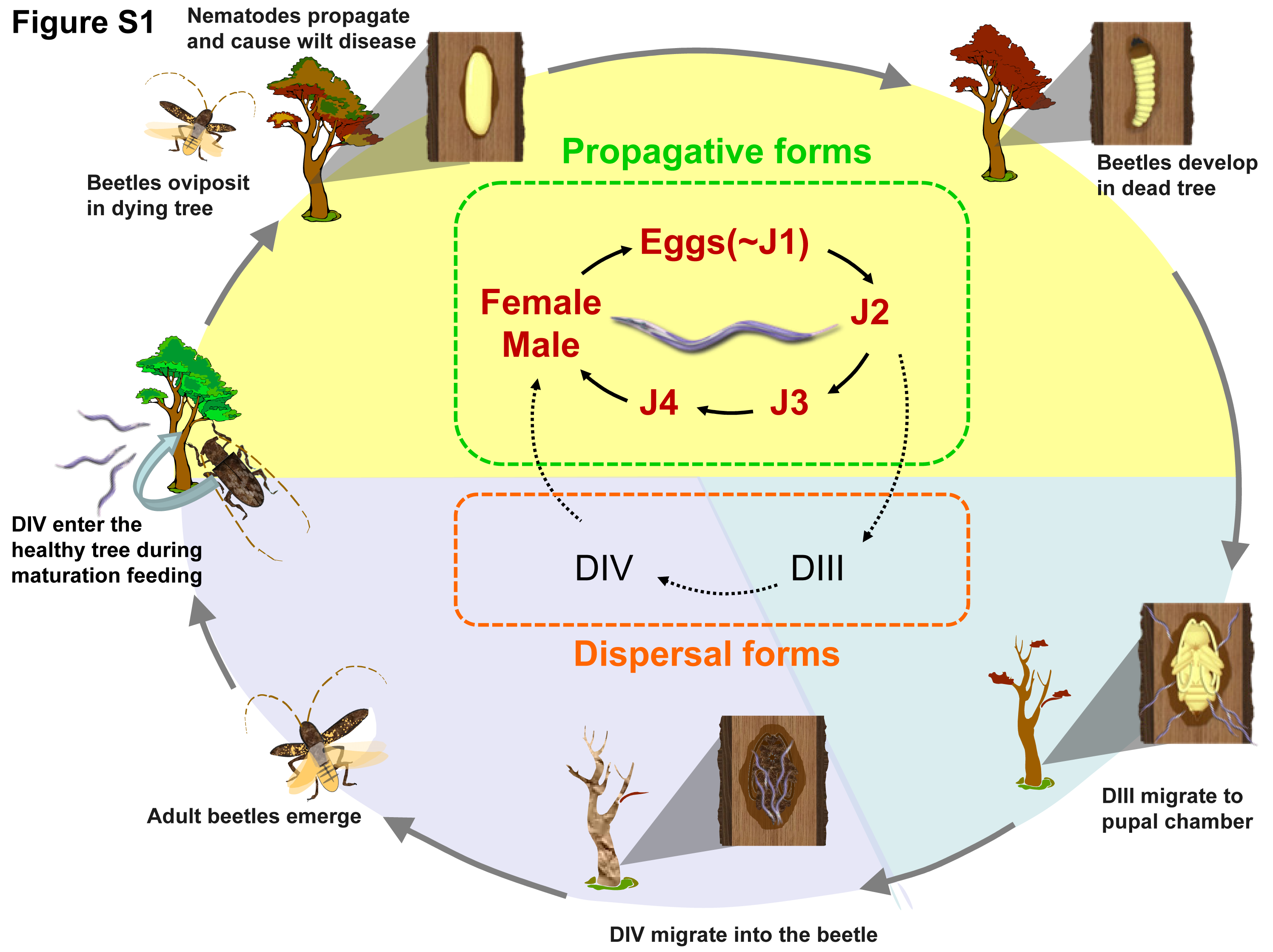

Supplement: Figure S1 — Life cycle of Bursaphelenchus xylophilus . The black arrows show B. xylophilus development cycle. The solid black arrows and dashed black arrows show the propagative cycle in pine trees (propagative forms) and that for transmission to new host trees by beetle vectors (dispersal forms), respectively. After invading healthy trees the forth-stage dispersal juvenile (DIV) of B. xylophilus molts to become the adult, and the propagative forms of B. xylophilus feed on the parenchymal cells in the resin canals. B. xylophilus is also able to feed on fungal growth after the plant cells are dead. (TIF) [file pone.0067377.s001.tif]

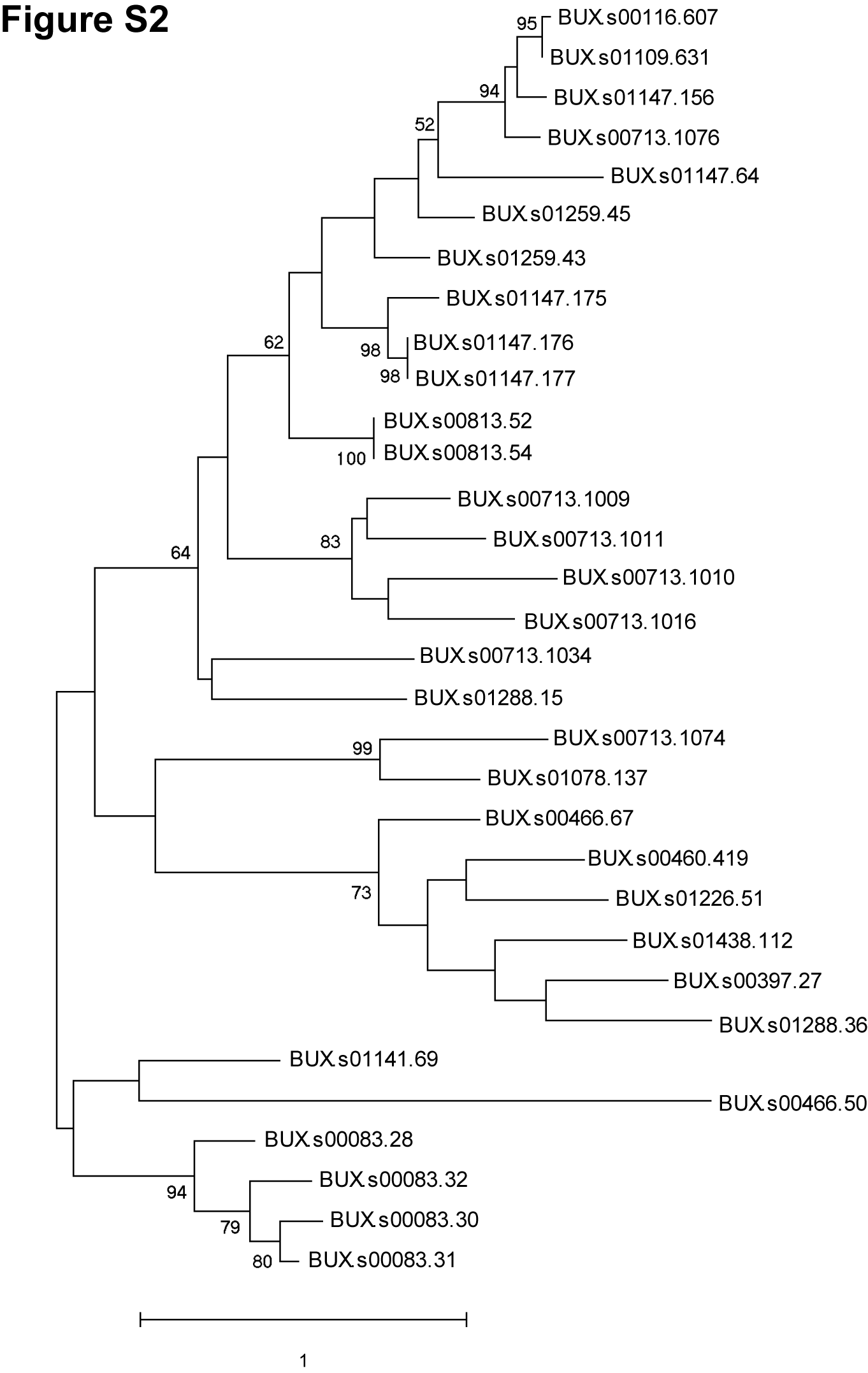

Supplement: Figure S2 — Phylogenetic relationships of the C1A (papain) family of cysteine peptidase secreted from Bursaphelenchus xylophilus . A multiple alignment of 205 aa was analyzed by Muscle and the phylogenetic tree was built using the maximum likelihood method in MEGA5 based on the JTT model with 1,000 bootstrap replicates. Bootstrap values more than 50% were shown in the tree. Four proteins (BUX.s00713.538, BUX.s0813.53, BUX.s0983.4, and BUX.s01063.86) with short lengths or with long branches in the preliminary tree were removed from the analysis. The scale bar indicates number of amino acid changes per site. (TIF) [file pone.0067377.s002.tif]

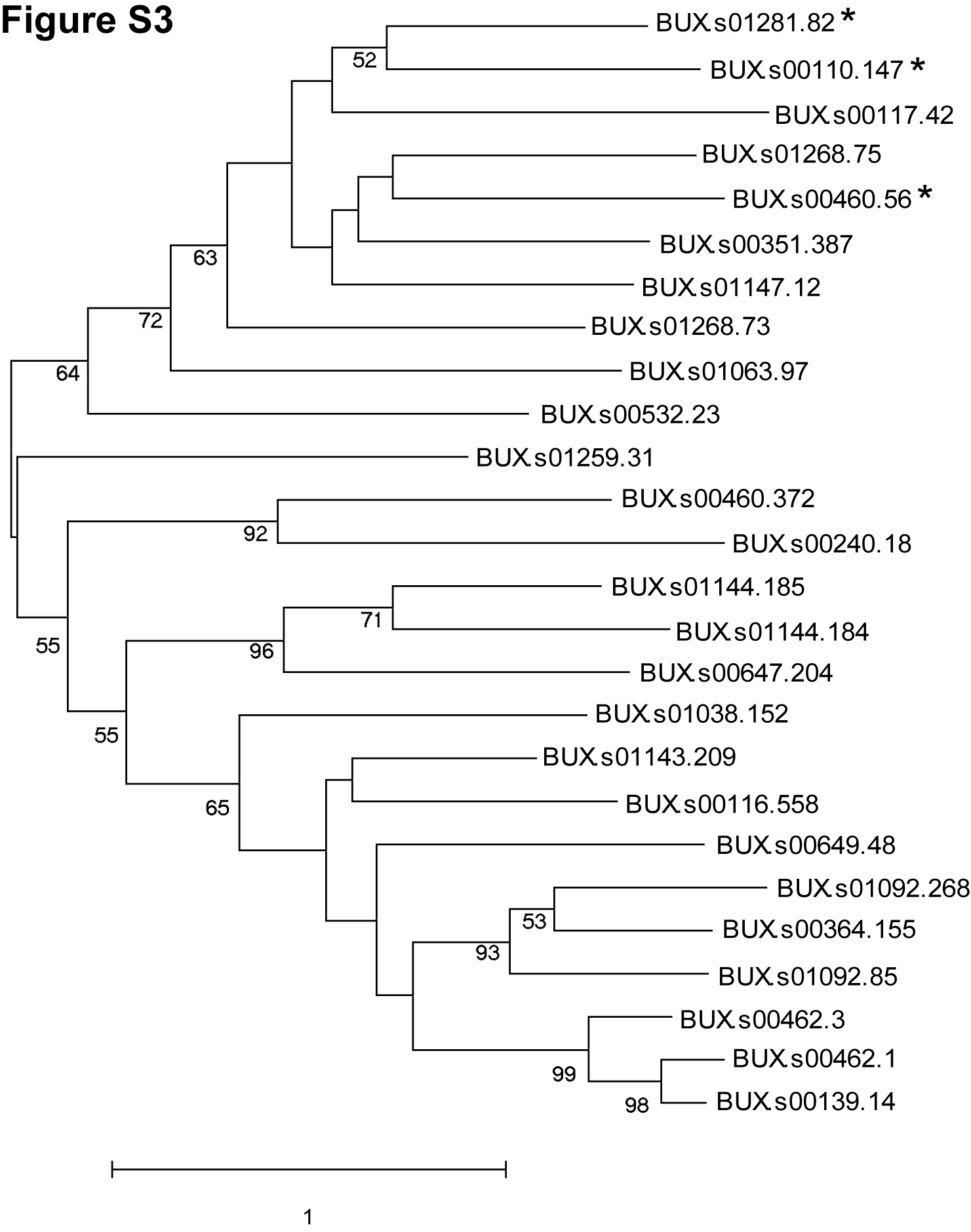

Supplement: Figure S3 — Phylogenetic relationships of the A1A (pepsin) family of aspartic peptidase secreted from Bursaphelenchus xylophilus . A multiple alignment of 338 aa was analyzed by Muscle and the phylogenetic tree was built using the maximum likelihood method in MEGA5 based on the JTT model with 1,000 bootstrap replicates. Bootstrap values more than 50% were shown in the tree. Five proteins (BUX.c03104.1, BUX.s01038.154, BUX.s01038.155, BUX.s00460.238, and BUX.s01150.38) with short lengths or with long branches in the preliminary tree were removed from the analysis. The scale bar indicates number of amino acid changes per site. *HGT indicates that the protein was acquired from other organism via horizontal gene transfer. These were selected based on the data of Kikuchi et al. [26]. (TIF) [file pone.0067377.s003.tif]

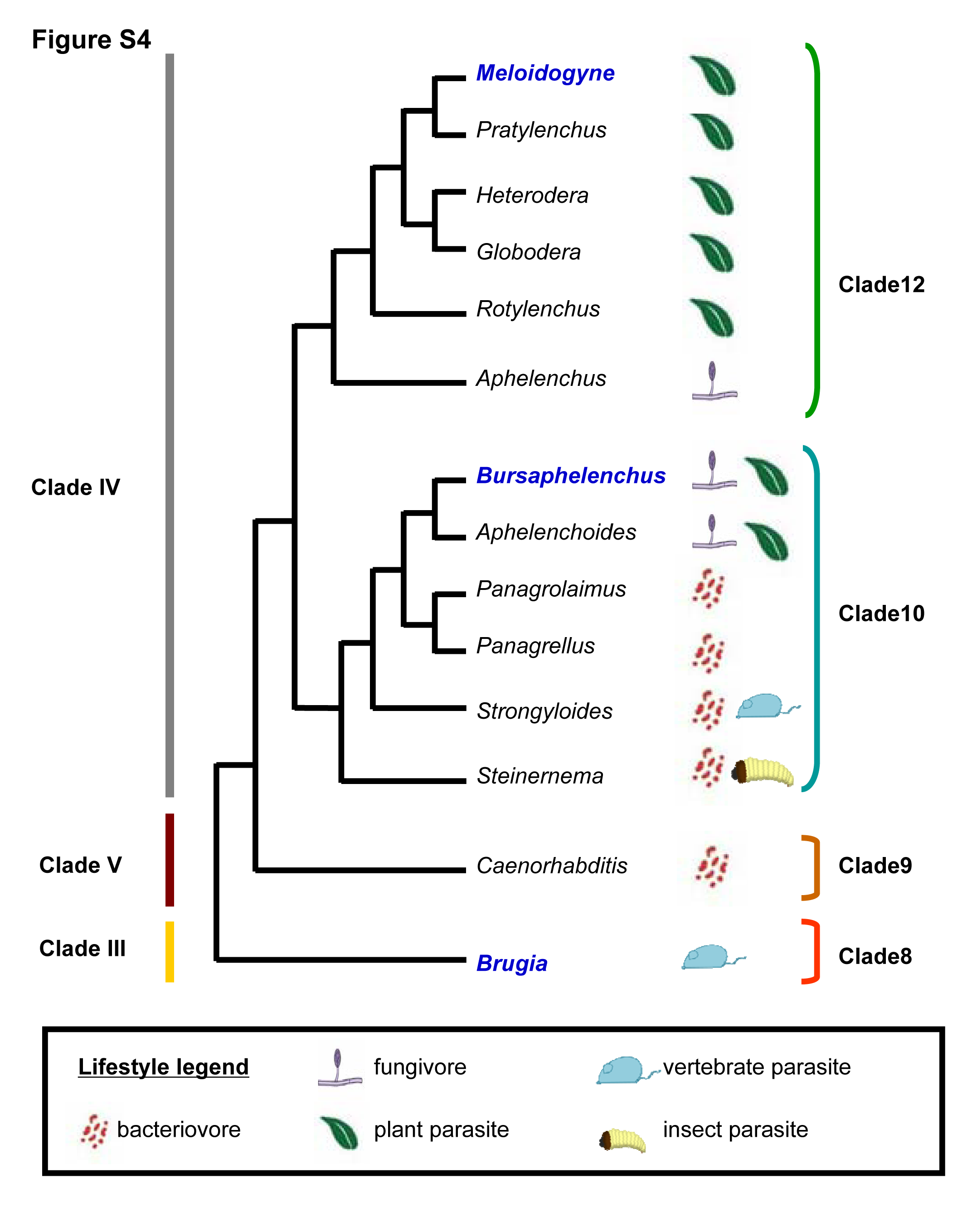

Supplement: Figure S4 — Schematic representation of the evolution of plant parasitism and the phylogenetic relationships of nematodes. The figure is adapted from Blaxter et al. [41] (major clades) and van Megen et al. [42] (minor clades). (TIF) [file pone.0067377.s004.tif]
